# Meta-analysis of the association between *NLRP1* polymorphisms and the susceptibility to vitiligo and associated autoimmune diseases

**DOI:** 10.18632/oncotarget.21165

**Published:** 2017-09-22

**Authors:** Juan Li, Min Yan, Yuan Zhang, Chao Feng, Huicong Wang, Cuiyu Wang, Li Sun

**Affiliations:** ^1^ Department of Dermatology, Central Hospital of Shengli Oil Field, Shandong, People's Republic of China

**Keywords:** NLRP1, vitiligo, autoimmune disease, polymorphism, meta-analysis

## Abstract

Genetic variants are linked to vitiligo and associated autoimmune diseases. We performed a meta-analysis to evaluate the effects of the rs12150220, rs2670660, and rs6502867 polymorphisms within the human NLR Family Pyrin Domain Containing 1 (*NLRP1*) gene. We initially identified 1,306 candidate articles through literature searches of Pubmed, WOS, Embase, CNKI, WANFANGI, Ovid, Scopus, and Cochrane in July 2017. After strict screening, we included 19 eligible case-control studies, and analyzed the data using Stata/SE 12.0 software. No difference between vitiligo cases and controls was detected for *NLRP1* rs12150220, rs2670660, or rs6502867 under most genetic models [*P*_association_ (*P* value of association test) > 0.05). With regard to vitiligo-associated autoimmune diseases, like Addison's disease, type 1 diabetes, or systemic lupus erythematosus, a decreased risk was detected for rs12150220 in the Caucasian subgroup under all models [*P*_association_ < 0.05, odds ratio (OR) < 1]. No relationships were observed for other polymorphisms, including rs2670660, rs6502867, and the “A-A, G-T, G-A, A-T” haplotypes of rs2670660/rs12150220 (*P*_association_ > 0.05). This meta-analysis demonstrates that within the Caucasian population, the NLRP1 rs12150220 polymorphism may correlate with a decreased risk of vitiligo-associated autoimmune diseases, especially autoimmune Addison's disease, type 1 diabetes, or systemic lupus erythematosus.

## INTRODUCTION

Vitiligo is a complicated autoimmune disorder characterized by skin depigmentation from progressively abnormal melanocytes [1, 2]. There are several vitiligo-associated autoimmune diseases, such as autoimmune thyroid disease (AITD), systemic sclerosis (SSc), juvenile idiopathic arthritis (JIA), Vogt-Koyanagi-Harada (VKH), autoimmune Addison's disease (AAD), type 1 diabetes (TID), rheumatoid arthritis (RA), psoriasis, pernicious anemia, alopecia areata, and systemic lupus erythematosus (SLE) [3–7]. Predisposition to vitiligo and its associated autoimmune diseases may be related to single nucleotide polymorphisms (SNPs) of genes, such as rs12150220, rs2670660, rs6502867, and rs8182352 of the *NLRP1* gene on chromosome 17p13.2, which is also named *NALP1* (NACHT, LRR, and PYD Domains-Containing Protein 1) [8–12]. NLRP1 protein is a member of the nucleotide oligomerization domain-like receptors (NLRs) family and regulates inflammasome activation, cellular apoptosis, innate immune system [13, 14], and some inflammatory disorders or autoimmune diseases [15]. Here, we studied the associations between rs12150220, rs2670660, and rs6502867 of *NLRP1* gene and the risks of vitiligo or associated autoimmune diseases via meta-analysis. The possible role of s2670660/rs12150220 haplotypes was also analyzed.

## RESULTS

### Study inclusion

**Figure 1:**
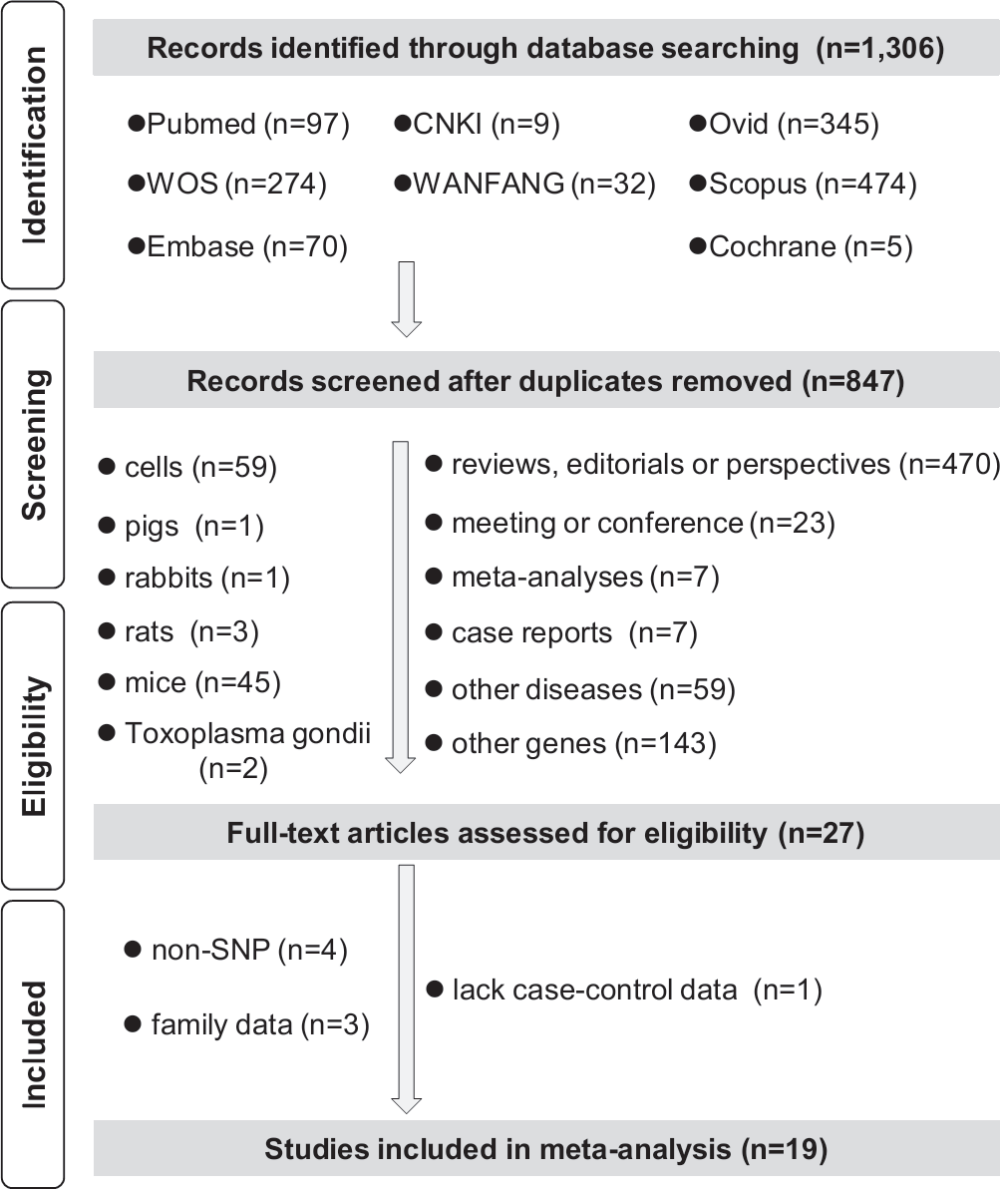
Flow chart of literature identification

We performed our meta-analyses in accordance with the guidelines of Preferred Reporting Items for Systematic Reviews and Meta-Analyses (PRISMA) [16] and strict inclusion/exclusion criteria. The checklist of PRISMA 2009 was shown in Supplementary Table 1. Initially, 1,306 candidate articles were retrieved from eight online databases: Pubmed (*n* = 97), Web of Science (WOS, *n* = 274), ExcerptaMedica Database (Embase, *n* = 70), China National Knowledge Infrastructure (CNKI, *N* = 9), WANFANG (*n* = 32), Ovid (*n* = 345), Scopus (*n* = 474), and Cochrane (*n* = 5). 459 duplicate articles were then removed. 820 articles were excluded after further screening. As indicated in PRISMA-based flow chart of Figure 1, we removed a number of 470 articles with the type of review, editorial or perspective, 23 articles with the abstracts of meeting or conference, 14 articles of meta-analyses, case reports. We also found that 59 articles contained the data of other diseases, 143 articles focused on other genes. Other articles involving the data of cells (*n* = 59), pigs (*n* = 1), rabbits (*n* = 1), rats (*n* = 3), mice (*n* = 45), Toxoplasma gondii (*n* = 2) were excluded as well. Then, eight articles were excluded for the reasons of non-SNP (*n* = 4), family data (*n* = 3) or lack of case-control data (*n* = 1). Nineteen eligible case-control studies were included in our final meta-analysis [4, 12, 17–33]. Three articles were published in Chinese, and the other sixteen articles were written in English. Study characteristics are in Supplementary Table 2, and all studies are have sufficient method quality [Supplementary Table 3, all Newcastle-Ottawa scale (NOS) score > 5].

### No overall relationship observed between NLRP1 rs12150220, rs2670660, rs6502867, and vitiligo risks

To study the association between rs12150220, rs2670660, and rs6502867 *NLRP1* polymorphisms and vitiligo risk, 563 cases and 1,351 controls from three Asian case-control studies underwent Mantel-Haenszel analysis of rs12150220. No between-study heterogeneities in any genetic models (Cochrane's Q statistic, *P*_heterogeneity_ > 0.1 and I^2^ test, I^2^ < 50%) led us to utilize fixed-effects model (Table 1). We observed an increased vitiligo risk in the case group in the models of AT vs. AA (Table 1, *P*_association_= 0.011, OR = 1.32), AT+TT vs. AA (*P*_association_ = 0.014, OR = 1.29), but not other models (all *P*_association_ > 0.05), compared with control group.

**Table 1:** Pooled analyses of the association between NLRP1 polymorphisms and susceptibility to vitiligo

SNP	Models	M	I^2^	*P*_heterogeneity_	Stratification	case/control (N)	OR [95% CI]	*P*_association_
**rs12150220**	T vs. A	Fixed	33.5%	0.222	overall/ Asian	563/1,351 (3)	1.17 [1.00~1.38]	0.057
	TT vs. AA	Fixed	0.0%	0.470	overall/ Asian	563/1,351 (3)	1.55 [0.77~1.72]	0.492
	AT vs. AA	Fixed	0.0%	0.896	overall/ Asian	563/1,351 (3)	1.32 [1.07~1.64]	0.011
	AT+TT vs. AA	Fixed	0.0%	0.861	overall/ Asian	563/1,351 (3)	1.29 [1.05~1.59]	0.014
	TT vs. AA+AT	Fixed	40.3%	0.187	overall/ Asian	563/1,351 (3)	0.97 [0.66~1.42]	0.866
	carrier T vs. A	Fixed	0.0%	0.513	overall/ Asian	563/1,351 (3)	1.15 [0.95~1.38]	0.149
**rs2670660**	G vs. A	Random	84.6%	< 0.001	overall	719/1,534 (5)	1.26 [0.84~1.90]	0.258
			88.2%	< 0.001	Asian	653/1,441(4)	1.19 [0.72~1.96]	0.491
			88.2%	< 0.001	*P*_HWE_ > 0.05	693/1,473 (4)	1.20 [0.75~1.90]	0.452
	GG vs. AA	Random	81.7%	< 0.001	overall	719/1,534 (5)	1.62 [0.73~3.59]	0.234
			85.9%	< 0.001	Asian	653/1,441(4)	1.40 [0.53~3.73]	0.500
			86.2%	< 0.001	*P*_HWE_ > 0.05	693/1,473 (4)	1.47 [0.58~3.71]	0.414
	AG vs. AA	Random	76.3%	0.002	overall	719/1,534 (5)	1.49 [0.88~2.53]	0.142
			78.6%	0.003	Asian	653/1,441 (4)	1.30 [0.73~2.32]	0.368
			80.7%	0.001	*P*_HWE_ > 0.05	693/1,473 (4)	1.37 [0.77~2.43]	0.289
	AG+GG vs. AA	Random	84.2%	< 0.001	overall	719/1,534 (5)	1.53 [0.84~2.81]	0.165
			86.9%	< 0.001	Asian	653/1,441 (4)	1.35 [0.68~2.66]	0.395
			87.6%	< 0.001	*P*_HWE_ > 0.05	693/1,473 (4)	1.40 [0.72~2.74]	0.327
	GG vs. AA+AG	Random	64.4%	0.024	overall	719/1,534 (5)	1.25 [0.77~2.01]	0.370
			72.9%	0.011	Asian	653/1,441(4)	1.20 [0.64~2.24]	0.573
			73.2%	0.011	*P*_HWE_ > 0.05	693/1,473 (4)	1.21 [0.68~2.13]	0.513
	carrier G vs. A	Random	56.5%	0.056	overall	719/1,534 (5)	1.18 [0.89~1.56]	0.262
			66.9%	0.028	Asian	653/1,441(4)	1.13 [0.80~1.61]	0.481
			66.8%	0.029	*P*_HWE_ > 0.05	693/1,473 (4)	1.14 [0.83~1.58]	0.420
**rs6502867**	C vs. T	Random	80.5%	< 0.001	overall	719/1,534 (5)	1.06 [0.70~1.61]	0.768
			64.7%	0.037	Asian	653/1,441(4)	1.29 [0.93~1.80]	0.130
			85.1%	< 0.001	*P*_HWE_ > 0.05	693/1,473 (4)	1.05 [0.65~1.70]	0.831
	CC vs. TT	Random	65.3%	0.021	overall	719/1,534 (5)	1.30 [0.62~2.72]	0.490
			52.1%	0.099	Asian	653/1,441(4)	1.73 [0.92~3.26]	0.397
			71.0%	0.016	*P*_HWE_ > 0.05	693/1,473 (4)	1.40 [0.64~3.07]	0.397
	TC vs. TT	Random	73.6%	0.004	overall	719/1,534 (5)	1.04 [0.64~1.69]	0.873
			55.7%	0.080	Asian	653/1,441(4)	1.27 [0.85~1.90]	0.238
			79.9%	0.002	*P*_HWE_ > 0.05	693/1,473 (4)	0.96 [0.55~1.69]	0.901
	TC+CC vs. TT	Random	80.1%	< 0.001	overall	719/1,534 (5)	1.07 [0.63~1.81]	0.805
			65.7%	0.033	Asian	653/1,441(4)	1.34 [0.87~2.07]	0.186
			85.1%	< 0.001	*P*_HWE_ > 0.05	693/1,473 (4)	1.01 [0.55~1.87]	0.963
	CC vs. TT+TC	Fixed	37.7%	0.170	overall	719/1,534 (5)	1.59 [1.21~2.09]	0.001
			15.1%	0.316	Asian	653/1,441(4)	1.69 [1.27~2.24]	< 0.001
			41.1%	0.165	*P*_HWE_ > 0.05	693/1,473 (4)	1.63 [1.24~2.16]	0.001
	carrier C vs. T	Random	54.7%	0.065	overall	719/1,534 (5)	1.07 [0.79~1.46]	0.653
			10.8%	0.339	Asian	653/1,441(4)	1.26 [1.04~1.53]	0.021
			65.9%	0.032	*P*_HWE_ > 0.05	693/1,473 (4)	1.05 [0.79~1.46]	0.782

SNP, Single Nucleotide Polymorphism; HWE, Hardy-Weinberg equilibrium; *P*_heterogeneity_, *P* value of Cochrane's Q statistic for the assessment of heterogeneity; M, statistical model; OR, odds ratio; CI, confidence interval; *P*_association_, *P* value of association test;

N, Number of included case-control studies.

Five case-control studies containing 719 cases and 1,534 controls were included for the rs2670660 meta-analysis. Negative results were observed in the overall and subgroup analysis of Asian, *P*_HWE_ > 0.05 (Table 1, all *P*_association_ > 0.05), based on random-effect model (Table 1, all *P*_heterogeneity_ < 0.1 and I^2^ > 50%). For rs6502867, the fixed-effects model was applied for CC vs. TT+TC (Table 1, *P*_heterogeneity_ = 0.170 and I^2^ = 37.7%), and random-effects model was applied for others (all *P*_heterogeneity_ < 0.1 and I^2^ > 50%). An increased vitiligo risk was only observed in CC vs. TT+TC models (Table 1, *P*_association_< 0.05, OR > 1), but not any other model (all *P*_association_> 0.05). Begg's test and Egger's test further excluded potential large publication bias (Supplementary Table 4, all *P*_Begg_ > 0.05, *P*_Egger_ > 0.05). All these results failed to provide strong evidence regarding an association between *NLRP1* rs12150220, rs2670660, rs6502867, and vitiligo risk.

### NLRP1 rs12150220 SNP may lower risk to vitiligo-associated autoimmune diseases in caucasians

As shown in Table 2, nineteen case-control studies (7,361 cases/28,722 controls) were included for the meta-analysis of the association between *NLRP1* rs12150220 and vitiligo-associated autoimmune diseases risk. A fixed-effects model was used for the models of AT vs. AA (Supplementary Table 5, *P*_heterogeneity_ = 0.213 and I^2^ = 19.8%) and carrier T vs. A (*P*_heterogeneity_ = 0.828 and I^2^ = 0.0%), whereas random-effects model was applied for others (all *P*_heterogeneity_ < 0.1). Pooled analysis indicated a decreased risk of case group in the models of T vs. A (*P*_association_= 0.003, OR = 0.91), TT vs. AA (*P*_association_= 0.004, OR = 0.84), AT vs. AA (*P*_association_< 0.001, OR = 0.89), AT+TT vs. AA (*P*_association_= 0.002, OR = 0.88), TT vs. AA+AT (*P*_association_ = 0.019, OR = 0.89), and carrier T vs. A (*P*_association_ =0.004, OR = 0.94) (Table 2). Similar results existed in the Caucasian subgroup analysis (all *P*_association_< 0.05, OR < 1).

**Table 2:** Pooled analyses of the association between NLRP1 rs12150220 polymorphisms and susceptibility to vitiligo-associated autoimmune diseases

Models	Stratification	case/control (N)	OR [95% CI]	*P*_association_
T vs. A	overall	7,361/28,722 (19)	0.91 [0.86~0.91]	**0.003**
	Asian	708/876 (4)	1.20 [0.92~1.56]	0.187
	Caucasian	6,493/27,338 (12)	0.90 [0.85~0.94]	**< 0.001**
	AITD	339/308 (3)	1.33 [1.07~1.66]	**0.012**
	AAD	753/5,700 (3)	0.78 [0.70~0.87]	**< 0.001**
	TID	2,568/5,892 (4)	0.89 [0.82~1.02]	**0.005**
	SLE	386/3,493 (3)	0.80 [0.68~0.95]	**0.013**
TT vs. AA	overall	563/1,351 (18)	0.84 [0.74~0.94]	**0.004**
	Asian	339/308 (3)	1.69 [1.07~2.68]	**0.025**
	Caucasian	6,493/27,338 (12)	0.80 [0.71~0.90]	**< 0.001**
	AITD	339/308 (3)	1.69 [1.07~2.68]	**0.025**
	AAD	753/5,700 (3)	0.57 [0.45~0.72]	**< 0.001**
	TID	2,568/5,892 (4)	0.78 [0.68~0.89]	**< 0.001**
	SLE	386/3,493 (3)	0.69 [0.49~0.98]	**0.038**
AT vs. AA	overall	7,361/28,722 (19)	0.89 [0.83~0.94]	**< 0.001**
	Asian	708/876 (4)	1.22 [0.93~1.61]	0.149
	Caucasian	6,493/27,338 (12)	0.87 [0.81~0.93]	**< 0.001**
	AITD	339/308 (3)	1.62 [1.13~2.31]	**0.008**
	AAD	753/5,700 (3)	0.87 [0.73~1.04]	0.124
	TID	2,568/5,892 (4)	0.86 [0.77~0.95]	**0.005**
	SLE	386/3,493 (3)	0.74 [0.57~0.96]	**0.021**
AT+TT vs. AA	overall	7,361/28,722 (19)	0.88 [0.81~0.96]	**0.002**
	Asian	708/876 (4)	1.22 [0.93~1.61]	0.193
	Caucasian	6,493/27,338 (12)	0.87 [0.81~0.93]	**< 0.001**
	AITD	339/308 (3)	1.62 [1.13~2.31]	**0.004**
	AAD	753/5,700 (3)	0.87 [0.73~1.04]	**0.003**
	TID	2,568/5,892 (4)	0.86 [0.77~0.95]	0.076
	SLE	386/3,493 (3)	0.74 [0.57~0.96]	**0.009**
TT vs. AA+AT	overall/ Asian	563/1,351 (18)	0.89 [0.81~0.98]	**0.019**
	Asian	339/308 (3)	1.26 [0.84~1.88]	0.265
	Caucasian	6,493/27,338 (12)	0.87 [0.78~0.97]	**0.015**
	AITD	339/308 (3)	1.26 [0.84~1.88]	0.265
	AAD	753/5,700 (3)	0.59 [0.43~0.80]	**0.001**
	TID	2,568/5,892 (4)	0.87 [0.77~0.98]	**0.017**
	SLE	386/3,493 (3)	0.81 [0.59~1.12]	0.201
carrier T vs. A	overall/ Asian	7,361/28,722 (19)	0.94 [0.90~0.98]	**0.004**
	Asian	708/876 (4)	1.10 [0.88~1.37]	0.390
	Caucasian	6,493/27,338 (12)	0.93 [0.89~0.97]	**0.002**
	AITD	339/308 (3)	1.22 [0.94~1.57]	0.130
	AAD	753/5,700 (3)	0.85 [0.75~0.96]	**0.010**
	TID	2,568/5,892 (4)	0.92 [0.85~1.07]	**0.038**
	SLE	386/3,493 (3)	0.86 [0.70~1.04]	0.122

AITD, autoimmune thyroid disease; AAD, Autoimmune Addison's disease; TID, Type 1 diabetes;

SLE, Systemic lupus erythematosus; OR, odds ratio; CI, confidence interval;

*P*_association_, *P* value of association test; N, Number of included case-control studies.

**Figure 2:**
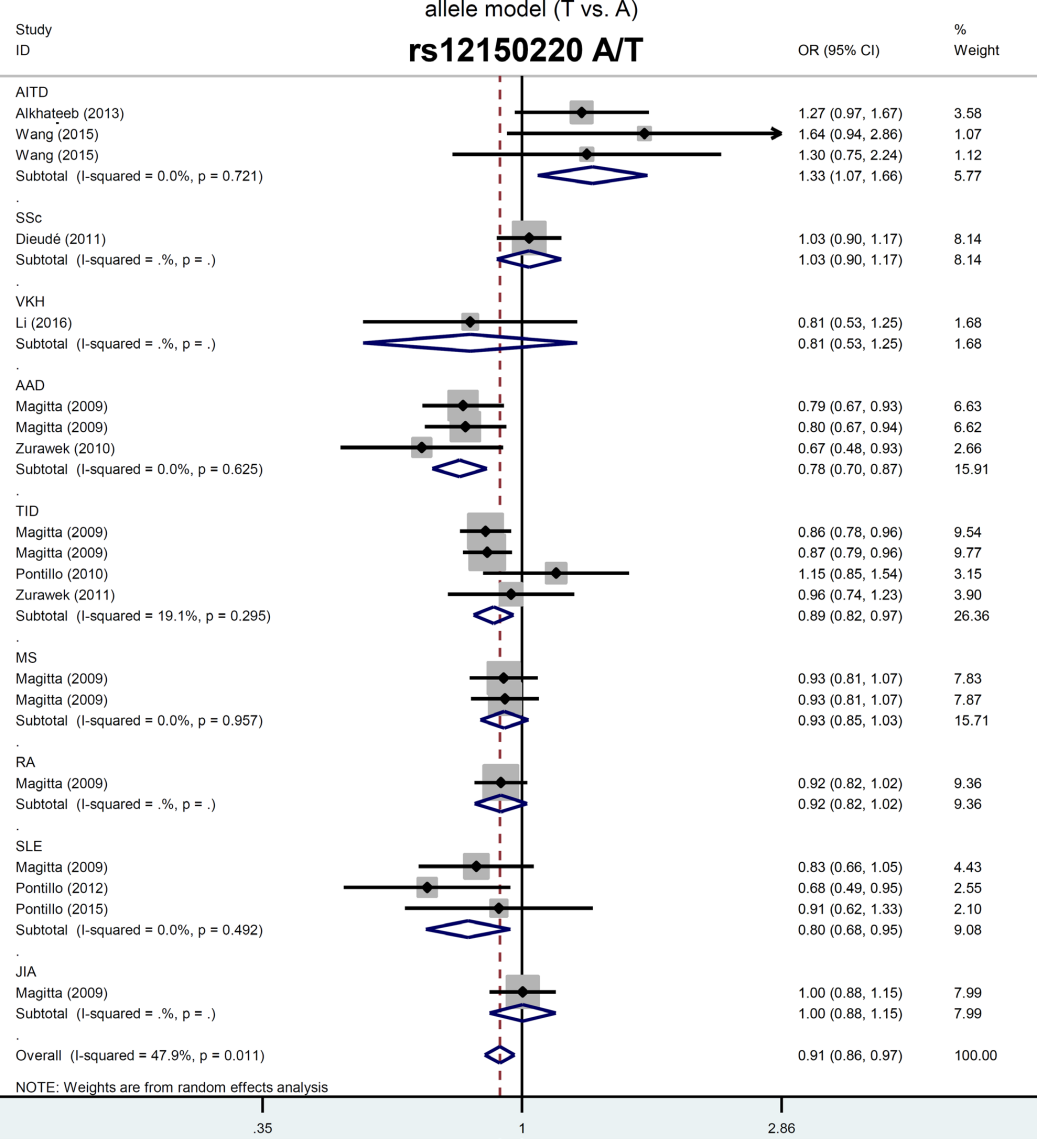
Forest plots of stratified analyses by disease type for *NLRP1* rs12150220, and susceptibility to vitiligo-associated autoimmune diseases under allele model

**Figure 3:**
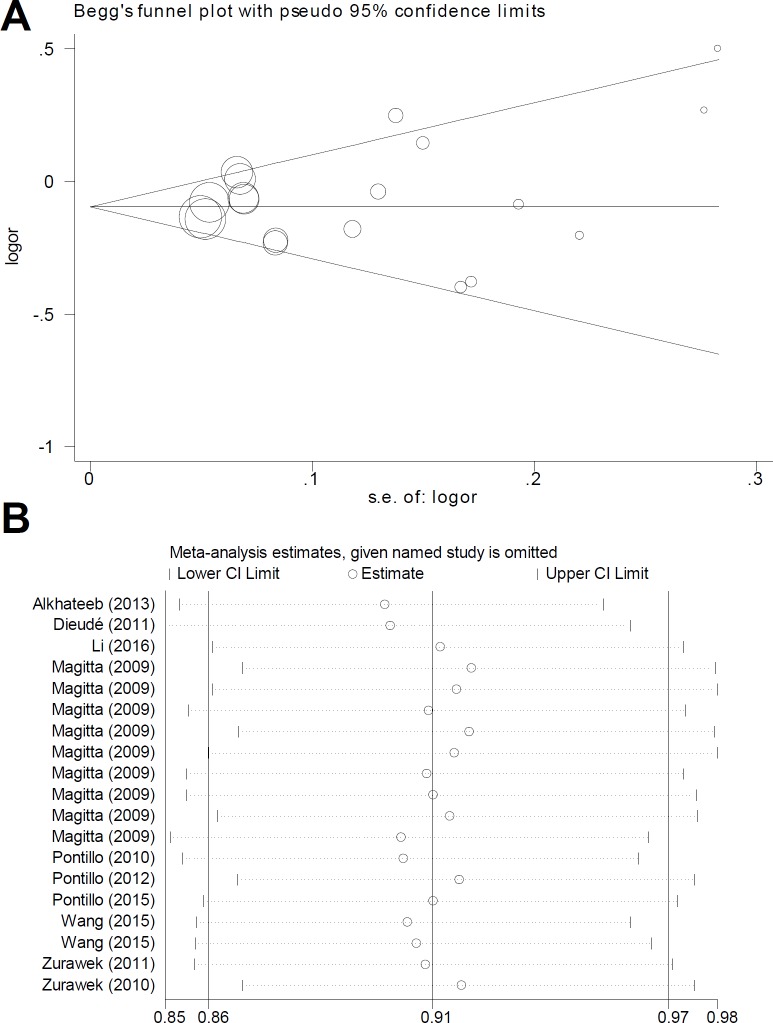
Begg's funnel plot and sensitivity analysis for the meta-analysis of the association between *NLRP1* rs12150220 and susceptibility to vitiligo-associated autoimmune diseases under allele model (**A**) Begg's funnel plot; (**B**) Sensitivity analysis.

Stratification analyses by specific disease type were also performed under every genetic model (Table 2), and forest plots were presented in Figure 2 and Supplementary Figures 1–5. An increased AITD risk was observed in the models of T vs. A, TT vs. AA, AT vs. AA, AT+TT vs. AA (all *P*_association_ < 0.05, OR > 1), but not TT vs. AA+AT (*P*_association_ = 0.265), and carrier T vs. A (*P*_association_ = 0.130). We detected a decreased SLE risk under all genetic models (all *P*_association_ > 0.05, OR < 1), but not the model of TT vs. AA+AT (*P*_association_ = 0.201) or carrier T vs. A (*P*_association_ = 0.122). A similar difference was detected in the ADD or TID case and control group under most of the genetic models (Table 2, all *P*_association_ < 0.05, OR < 1). Potential large publication was excluded by our Begg's test and Egger's test (Figure 3A, Supplementary Table 5, all *P*_Begg_ > 0.05, *P*_Egger_ > 0.05). Sensitivity analysis data (Figure 3B for allele model; data for other models not shown) suggested the stability of our results. The minor T allele of *NLRP1* rs12150220 is potentially associated with the reduced risk of vitiligo-associated autoimmune diseases in the Caucasian population, especially SLE, ADD, and TID.

### NLRP1 rs2670660 and rs6502867 may be not linked to vitiligo-associated autoimmune diseases risk

Regarding the Mantel-Haenszel statistic of rs2670660, a fixed-effects model was used in the AG vs. AA model (Supplementary Table 5, *P*_heterogeneity_ = 0.215 and I^2^ = 24.8%) and carrier G vs. A (*P*_heterogeneity_ = 0.514 and I^2^ = 0.0%), and random-effects model was used for others (all *P*_heterogeneity_ < 0.1). No difference was observed in the case and control groups for the overall meta-analysis and stratification analysis under most of genetic models (Supplementary Table 6, *P*_association_ > 0.05). A slight publication bias was found in the models of AG vs. AA (Supplementary Table 5, *P*_Begg_= 0.032, *P*_Egger_ = 0.034) and AG+GG vs. AA (*P*_Begg_= 0.012, *P*_Egger_ = 0.027), but not others (Supplementary Figure 6, Supplementary Table 5, all *P*_Begg_ > 0.05, *P*_Egger_ > 0.05). Similar results were observed in the rs6502867 meta-analysis (Supplementary Figure 7, Supplementary Tables 5, 7). Overall, rs2670660 and rs6502867 NLRP1 polymorphisms seem not to be associated with the risk of vitiligo-associated autoimmune diseases.

### NLRP1 rs2670660/rs12150220 haplotype have no vitiligo-associated autoimmune diseases risks

Finally, we measured the association between *NLRP1* rs2670660/rs12150220 haplotype and the risk of vitiligo-associated autoimmune diseases. Five case-control studies were included, and four *NLRP1* rs2670660/rs12150220 haplotypes were selected: A-A, G-T, G-A, A-T. A fixed-effects model was used for G-T haplotype (*P*_heterogeneity_ = 0.634 and I^2^ = 0.0%), and a random-effects model was used for all others (all *P*_heterogeneity_ < 0.1 and I^2^ > 50.0%) (Supplementary Table 8). Mantel-Haenszel data showed no difference between the case and control groups, and large publication bias was excluded for all these meta-analyses (Supplementary Table 8, all *P*_association_> 0.05, *P*_Begg_ > 0.05, *P*_Egger_ > 0.05). Thus, *NLRP1* rs2670660/rs12150220 haplotypes are unlikely to be related to vitiligo-associated autoimmune diseases risks.

## DISCUSSION

Incongruent conclusions have been reported for the associations of three *NLRP1* SNPs (rs12150220, rs2670660, rs6502867) with vitiligo risk [12, 18, 20, 30]. Those same polymorphisms also yield contradictory conclusions on their correlation with vitiligo-associated autoimmune diseases [4, 24, 25, 31, 32]. Due to these disparate results, we performed a comprehensive meta-analysis to explore the association between *NLRP1* SNPs and vitiligo susceptibility, as well as vitiligo-associated autoimmune diseases.

We focused on the potential role of *NLRP1* variation in the risks of vitiligo and vitiligo-associated autoimmune diseases by systematically identifying all available relevant case-control studies. Literature was compiled from eight online databases: Pubmed, WOS, Embase, CNKI, WANFANGI, Ovid, Scopus, and Cochrane, with no publication date limitation. During the literature searches of Pubmed, WOS, Embase, CNKI, WANFANGI, and Cochrane, we sensed that less than 300 potential articles were acquired in each database, when using the search terms about “*NLRP1*” and “SNP”. For example, only 97 potential articles on the association between *NLRP1* polymorphisms and clinical diseases were retrieved from the database of Pubmed. Considering these limited literatures, and naming complexity of the vitiligo-associated autoimmune diseases, we thoroughly checked these original articles one by one, in order to prevent the possible filtration of targeting articles during the advanced search using more terms. Only three common polymorphic variants were selected, including rs12150220, rs2670660, and rs6502867. We found that most participants in the included case-control studies of vitiligo were enrolled from Asian regions, but that most data were published in English.

Our data did not provide evidence of an association between the *NLRP1* rs12150220, rs2670660, and rs6502867 genotypes and the risks of vitiligo in the overall population. We found that the major A allele of rs12150220 was positively correlated with high risk to vitiligo-associated autoimmune diseases, including autoimmune Addison's disease, type 1 diabetes, and systemic lupus erythematosus. This suggests the rare T allele may serve as a protective factor against those diseases. The rs12150220 polymorphism may attenuate abnormal NLRP1 protein activity or the related inflammation/apoptosis process in the pathogenesis of vitiligo-associated autoimmune diseases. In addition, we observed a negative association for the rs2670660, rs6502867, and “A-A, G-T, G-A, A-T” haplotypes of s2670660/rs12150220.

With all meta-analyses, limitations regarding sample size, SNP selection, disease features, and heterogeneity origins need acknowledged. There were limited case-control studies available for inclusion. For instance, meta-analysis of the association between *NLRP1* rs12150220 and vitiligo in the Asian population was performed on just three case-control studies from two articles [18, 20]. Subgroup analyses of other disease types also contained small sample sizes. For instance, only one case-control study was used for the association between rs12150220 and rheumatoid arthritis [4]. Although only three SNPs were selected for this study, due to the requirement of meta-analysis on the enrolled number of case-control studies (at least > = 3), other SNPs such as rs8182352 and rs4790797 should be analyzed when sufficient genotype frequency data becomes available. The joint effects of different SNPs, or more stratified meta-analyses by ethnic group, control source, and disease type need to be studied as well.

Not all patients included in the study presented with both vitiligo and a vitiligo-associated autoimmune disease. More data of cases suffering from both vitiligo and associated autoimmune diseases are required for more confident conclusions. Moreover, not all the other vitiligo-associated autoimmune diseases were analyzed. Some other vitiligo-associated autoimmune diseases, such as psoriasis, pernicious anemia and alopecia areata, were not investigated, owing to a lack of published case-control studies.

We observed high between-study heterogeneities when analyzing rs2670660 and rs6502867 in vitiligo under most genetic models. Subgroup data showed that ethnicity or HWE may not contribute to heterogeneity, and sensitivity analysis also failed to reveal a source for the heterogeneities (data not shown). In addition, there were high heterogeneities among studies of the association between rs2670660, rs6502867, and vitiligo-associated autoimmune diseases under the allele, homozygote, dominant, and recessive models. However, the relative low probability of heterogeneities exists in disease-based subgroup analysis, suggesting the complexity of different specific disease types is the possible cause of heterogeneity. In addition, we observe an increased risk trend for the autoimmune thyroid disease patients with the minor allele. The differences of etiology and clinical characteristics of vitiligo-associated autoimmune diseases should be fully considered. Unavailable data factors such as different genotyping assays, diagnostic criteria, or clinical features may also contribute to high between-study heterogeneity.

In sum, we identified a potential genetic relationship in the Caucasian population between the *NLRP1* rs12150220 polymorphism and a decreased susceptibility to autoimmune diseases, especially autoimmune Addison's disease, type 1 diabetes, or systemic lupus erythematosus. These autoimmune diseases were all tightly associated with vitiligo. However, we did not observe a strong association between *NLRP1* rs12150220, rs2670660, or rs6502867 and vitiligo risk, according to the currently very limited data. Similarly, rs2670660 and rs6502867 polymorphisms and rs2670660/rs12150220 haplotypes (A-A, G-T, G-A, A-T) within *NLRP1* appear to have no effect on the risk of vitiligo-associated autoimmune diseases. Given the fact of insufficient statistical power as stated above, more data was needed to confirm these statements, and further determine the role of *NLRP1* SNPs in the presence of vitiligo, or vitiligo together with autoimmune diseases.

## MATERIALS AND METHODS

### Literature identification

Pubmed, WOS, Embase, CNKI, WANFANGI, Ovid, Scopus, and Cochrane, were utilized for identifying candidate literature up to July 2017. Supplementary Table 9 shows the specific search terms used. Exclusion criteria: (a) duplicate articles; (b) reviews, editorials, or perspectives; (c) meeting or conference abstracts; (d) meta-analyses; (e) case reports; (f) articles containing cell, pig, rabbit, rat, mice or Toxoplasma gondii data; (g) lack of case-control data; (h) containing the data of non-SNP; (i) family disease. The included case-control studies contained sufficient allele and genotype frequencies of case/control group for meta-analysis, ethnicity, SNP, genotyping method, source of control, and Hardy-Weinberg equilibrium (HWE). All these were independently extracted by J. Li and M. Yan. The missing data were obtained through the corresponding author.

### Quality evaluation

Before statistical analysis, the quality of all these case-control studies was evaluated by the Newcastle-Ottawa scale (NOS) system, which is available at http://www.ohri.ca/programs/clinical_epidemiology/oxford.asp. Only high-quality studies (NOS > 5) were included in our meta-analysis [34, 35]. The discrepancies were resolved by the consensus from J. Li, M. Yan and L. Sun.

### Mantel-Haenszel analysis

To calculate the two-tailed *P* value of association (*P*_association_), OR (odds ratio), and 95% CI (95% confidence interval), Mantel-Haenszel analysis was performed on basis of six genetic models, namely allele (T vs. A for rs12150220; G vs. A for rs2670660; C vs. T for rs6502867), homozygote (TT vs. AA; GG vs. AA; CC vs. TT), heterozygote (AT vs. AA; AG vs. AA; TC vs. TT), dominant (AT+TT vs. AA; AG+GG vs. AA; TC+CC vs. TT), recessive (TT vs. AA+AT; GG vs. AA+AG; CC vs. TT+TC), or carrier (carrier T vs. A; carrier G vs. A; carrier C vs. T) models. *P*_association_ < 0.05 indicates correlation, and pooled OR > 1 suggests T allele of rs12150220 A/T, G allele of rs2670660 A/G, or C allele of rs6502867 T/C may act as a risk factor.

### Heterogeneity and publication bias

We assessed heterogeneity across case-control studies through both Cochrane's Q statistic and I^2^ test. When *P*_heterogeneity_ value of Cochrane's Q statistic > 0.1 and I^2^ values < 50.0% at the same time, heterogeneity across studies was excluded, and a fixed-effects model was used. Otherwise, a random-effects model was utilized. Subgroup analyses of specific disease type, ethnicity, or HWE, and leave-one-out sensitivity analyses were then performed. A symmetrical funnel plot, *P*_Begg_ > 0.05 and *P*_Egger_ > 0.05 ruled out publication bias. We performed chi-square test to measure the Hardy-Weinberg equilibrium of control group. All analysis was performed with STATA12.0 (College Station, TX, USA).

## SUPPLEMENTARY TABLES AND FIGURES








