# Supplementary material for: Meta-analysis of the association between NLRP1 polymorphisms and the susceptibility to vitiligo and associated autoimmune diseases
Source: Oncotarget. 2017 Sep 22;8(50):88179–88. doi: 10.18632/oncotarget.21165 (PMC5675702; doi:10.18632/oncotarget.21165)
Supplement: Supplementary file 1 [file oncotarget-08-88179-s001.pdf]

Meta-analysis of the association between *NLRP1* polymorphisms and the susceptibility to vitiligo and associated autoimmune diseases

SUPPLEMENTARY MATERIALS

Supplementary Table 1: PRISMA 2009 checklist. See Supplementary_Table_1

Supplementary Table 2: Characteristics of included case-control studies. See Supplementary_Table_2

Supplementary Table 3: NOS-based quality assessment

Number	First author	Year	NOS score	Term 1	Term 2	Term 3	Term 4	Term 5	Term 6	Term 7	Term 8	Term 9
**1**	**Alkhateeb**	2013	8	1	1	1	1	1	0	1	1	1
**2**	**Alkhateeb**	2010	8	0	1	1	1	1	1	1	1	1
**3**	**Dieudé**	2011	6	0	1	1	1	1	0	1	1	0
**4**	**Dwivedi**	2013	9	1	1	1	1	1	1	1	1	1
**5**	**Goh**	2017	7	0	1	1	1	1	1	0	1	1
**6**	**Hinks**	2013	7	0	1	1	1	1	0	1	1	1
**7**	**Horie**	2011	8	1	1	1	1	1	0	1	1	1
**8**	**Jin**	2007	8	1	1	1	1	0	1	1	1	1
**9**	**Li**	2016	8	1	1	1	1	1	1	1	1	0
**10**	**Magitta**	2009	7	1	1	1	1	1	0	1	1	0
**11**	**Pontillo**	2010	8	1	1	1	1	1	0	1	1	1
**12**	**Pontillo**	2012	8	1	1	1	1	1	0	1	1	1
**13**	**Pontillo**	2015	7	1	1	1	1	1	0	1	1	0
**14**	**Sui**	2012	8	1	1	1	1	1	1	1	1	0
**15**	**Sun**	2013	5	0	1	0	0	1	1	1	1	0
**16**	**Wang**	2015	9	1	1	1	1	1	1	1	1	1
**17**	**Xie**	2008	7	1	1	1	1	1	0	1	1	0
**18**	**Zurawek**	2011	8	0	1	1	1	1	1	1	1	1
**19**	**Zurawek**	2010	7	0	1	1	1	1	0	1	1	1

NOS, Newcastle-Ottawa scale; Term 1, Definition of cases; Term 2, Representativeness of cases; Term 3, Selection of controls; Term 4, Definition of controls; Term 5, Important factors of comparability; Term 6, Other factors of comparability; Term 7, secure record of exposure; Term 8, same genotyping method; Term 9, Non-response rate.

Supplementary Table 4: Publication bias assessment for vitiligo meta-analysis

SNP	Models	Begg's test		Egger's test	
		z	P_{Begg}	***t***	P_{Egger}
**rs12150220**	T vs. A	1.04	0.296	-2.88	0.213
	TT vs. AA	0.00	1.000	-1.51	0.372
	AT vs. AA	0.00	1.000	0.30	0.812
	AT+TT vs. AA	1.04	0.296	-6.13	0.103
	TT vs. AA+AT	0.00	1.000	-1.31	0.414
	carrier T vs. A	1.04	0.296	-3.46	0.179
**rs2670660**	G vs. A	0.24	0.806	-0.60	0.591
	GG vs. AA	0.73	0.462	-0.58	0.605
	AG vs. AA	-0.24	1.000	0.26	0.813
	AG+GG vs. AA	-0.24	1.000	0.04	0.973
	GG vs. AA+AG	0.24	0.806	-1.39	0.259
	carrier G vs. A	-0.24	1.000	-0.61	0.584
**rs6502867**	C vs. T	-0.24	1.000	-1.50	0.231
	CC vs. TT	-0.24	1.000	-1.29	0.289
	TC vs. TT	0.24	0.806	-1.16	0.330
	TC+CC vs. TT	0.24	0.806	-1.28	0.290
	CC vs. TT+TC	0.24	0.806	-1.37	0.263
	carrier C vs. T	0.24	0.806	-1.53	0.223

P_{Begg}, P value of Begg's test; P_{Egger}, P value of Egger's test.

Supplementary Table 5: Heterogeneity and publication bias assessment for meta-analysis of vitiligo-associated autoimmune diseases

SNP	Models	Heterogeneity			Begg's test		Egger's test	
		I^2	$P_{heterogeneity}$	M	*z*	P_{Begg}	*t*	P_{Begg}
**rs12150220**	T vs. A	47.9%	0.011	Random	0.49	0.624	0.71	0.489
	TT vs. AA	47.4%	0.014	Random	0.23	0.820	0.49	0.631
	AT vs. AA	19.8%	0.213	Fixed	1.33	0.184	1.99	0.063
	AT+TT vs. AA	36.8%	0.055	Random	1.12	0.263	1.62	0.123
	TT vs. AA+AT	36.9%	0.059	Random	0.00	1.000	-0.37	0.713
	carrier T vs. A	0.0%	0.828	Fixed	0.28	0.780	0.67	0.514
**rs2670660**	G vs. A	64.1%	0.003	Random	1.79	0.074	2.16	0.063
	GG vs. AA	94.1%	<0.001	Random	0.72	0.474	0.94	0.373
	AG vs. AA	24.8%	0.215	Fixed	2.15	**0.032**	2.56	**0.034**
	AG+GG vs. AA	49.4%	0.038	Random	2.50	**0.012**	2.71	**0.027**
	GG vs. AA+AG	57.4%	0.012	Random	1.25	0.210	1.39	0.202
	carrier G vs. A	0.514	0.0%	Fixed	1.79	0.074	2.18	0.061
**rs6502867**	C vs. T	0.0%	0.590	Fixed	2.63	**0.009**	-3.15	**0.005**
	CC vs. TT	0.0%	0.856	Fixed	1.11	0.266	-1.43	0.172
	TC vs. TT	16.3%	0.246	Fixed	1.30	0.194	-1.81	0.086
	TC+CC vs. TT	5.8%	0.384	Fixed	2.02	**0.043**	-2.31	**0.032**
	CC vs. TT+TC	0.0%	0.770	Fixed	0.87	0.387	-1.34	0.199
	carrier C vs. T	0.0%	0.931	Fixed	2.99	**0.003**	-3.25	**0.004**

$P_{heterogeneity}$, *P* value of Cochrane's Q statistic for the assessment of heterogeneity; M, statistical model; *PBegg*, *P* value of Begg's test;
PEgger, *P* value of Egger's test.

Supplementary Table 6: Pooled analyses of the association between NLRP1 rs2670660 polymorphism and susceptibility to vitiligo-associated autoimmune diseases

Models	Stratification	case/control (N)	OR [95 % CI]	$P_{association}$
**G vs. A**	overall	3.732/8,978 (10)	1.12 [1.01~1.25]	**0.034**
	Caucasian	3,098/8,250 (6)	1.02 [0.96~1.09]	0.504
	$P_{HWE} > 0.05$	1,813/2,171 (7)	1.22 [1.02~1.47]	**0.029**
	$P_{HWE} < 0.05$	1,919/6,807 (3)	1.02 [0.94~1.09]	0.661
	TID	1,501/2,715 (3)	1.02 [0.93~1.11]	0.699
**GG vs. AA**	overall	3.732/8,978 (10)	1.08 [0.65~1.80]	0.762
	Caucasian	3,098/8,250 (6)	0.66 [0.37~1.19]	0.170
	$P_{HWE} > 0.05$	1,813/2,171 (7)	1.58 [0.99~2.53]	0.057
	$P_{HWE} < 0.05$	1,919/6,807 (3)	0.48 [0.22~1.06]	0.069
	TID	1,501/2,715 (3)	1.13 [0.83~1.54]	0.437
**AG vs. AA**	overall	3.732/8,978 (10)	1.05 [0.96~1.15]	0.284
	Caucasian	3,098/8,250 (6)	1.00 [0.91~1.11]	0.927
	$P_{HWE} > 0.05$	1,813/2,171 (7)	1.12 [0.96~1.29]	0.141
	$P_{HWE} < 0.05$	1,919/6,807 (3)	1.01 [0.90~1.14]	0.841
	TID	1,501/2,715 (3)	0.95 [0.83~1.10]	0.527
**AG+GG vs. AA**	overall	3.732/8,978 (10)	1.13 [0.99~1.30]	0.078
	Caucasian	3,098/8,250 (6)	1.02 [0.93~1.12]	0.728
	$P_{HWE} > 0.05$	1,813/2,171 (7)	1.28 [1.00~1.65]	0.053
	$P_{HWE} < 0.05$	1,919/6,807 (3)	1.02 [0.91~1.14]	0.750
	TID	1,501/2,715 (3)	0.98 [0.86~1.13]	0.790
**GG vs. AA+AG**	overall	3.732/8,978 (10)	1.18 [0.99~1.40]	0.071
	Caucasian	3,098/8,250 (6)	1.05 [0.94~1.17]	0.409
	$P_{HWE} > 0.05$	1,813/2,171 (7)	1.34 [1.01~1.76]	**0.040**
	$P_{HWE} < 0.05$	1,919/6,807 (3)	1.03 [0.88~1.20]	0.734
	TID	1,501/2,715 (3)	1.09 [0.92~1.28]	0.309
**carrier G vs. A**	overall	3.732/8,978 (10)	1.05 [0.98~1.12]	0.179
	Caucasian	3,098/8,250 (6)	1.01 [0.94~1.09]	0.707
	$P_{HWE} > 0.05$	1,813/2,171 (7)	1.10 [0.99~1.22]	0.070
	$P_{HWE} < 0.05$	1,919/6,807 (3)	1.01 [0.93~1.10]	0.803
	TID	1,501/2,715 (3)	1.01 [0.91~1.12]	0.849

HWE, Hardy-weinberg equilibrium; TID, Type 1 diabetes; OR, odds ratio; CI, confidence interval; *Passociation*, *P* value of Association Test; N, Number of included case-control studies.

Supplementary Table 7: Pooled analyses of the association between NLRP1 rs6502867 polymorphism and susceptibility to vitiligo-associated autoimmune diseases. See Supplementary_Table_7

Supplementary Table 8: Pooled analyses of the association between *NLRP1* rs2670660/rs12150220 haplotype and susceptibility to vitiligo-associated autoimmune diseases

haplotypes		Heterogeneity			case/control (N)	Association Test		Begg's test		Egger's test	
**rs2670660 A/G**	**rs12150220 A/T**	**I^2**	**$P_{heterogenity}$**	**M**		**OR [95 % CI]**	**$P_{association}$**	**z**	P_{Begg}	***t***	**P_{Egger}**
**A**	**A**	69.6%	0.011	Random	701/1,044 (5)	0.97 [0.65~1.43]	0.866	0.73	0.462	2.56	0.083
**G**	**T**	0.0%	0.634	Fixed	563/1,351 (5)	1.02 [0.82~1.28]	0.864	-0.24	1.000	-0.42	0.700
**G**	**A**	70.4%	0.009	Random	563/1,351 (5)	1.35 [0.81~2.25]	0.244	-0.24	1.000	-0.03	0.976
**A**	**T**	63.4%	0.027	Random	563/1,351 (5)	1.08 [0.58~2.04]	0.800	0.24	0.806	0.11	0.919

Pheterogeneity, *P* value of Cochrane's Q statistic; M, statistical model; N, Number of included case-control studies; OR, odds ratio; CI, confidence interval; *Passociation*, *P* value of Association Test; N, Number; P_{Begg}, *P* value of Begg's test; P_{Egger}, *P* value of Egger's test.

Supplementary Table 9: Search terms used for meta-analysis (up to July 2017)

Database	Step	Search terms	Number of articles
Pubmed	#1	"Polymorphism, Single Nucleotide"[Mesh]	**89,956**
	#2	(((((Nucleotide Polymorphism, Single) OR Nucleotide Polymorphisms, Single) OR Polymorphisms, Single Nucleotide) OR Single Nucleotide Polymorphisms) OR SNPs) OR Single Nucleotide Polymorphism	**117,052**
	#3	#2 OR #3	**117,052**
	#4	((((((((((NLRP1) OR NALP1) OR NLR Family Pyrin Domain Containing 1) OR (NACHT, LRR And PYD Domains-Containing Protein 1)) OR NACHT leucine-rich-repeat protein 1) OR death effector filament-forming Ced-4-like apoptosis) OR (NACHT, leucine rich repeat and PYD containing 1)) OR NLR family, pyrin domain containing 1) OR DEFCAP) OR DEFCAP-S) OR DEFCAP-L	**1,949**
	#5	#3 AND #4	**97**
WOS	#1	TOPIC: (Polymorphism, Single Nucleotide) OR TOPIC: (Nucleotide Polymorphism, Single) OR TOPIC: (Nucleotide Polymorphisms, Single) OR TOPIC: (Polymorphisms, Single Nucleotide) OR TOPIC: (Single Nucleotide Polymorphisms) OR TOPIC: (SNPs) OR TOPIC: (Single Nucleotide Polymorphism) OR TOPIC: (Mutation) OR TOPIC: (Mutations) OR TOPIC: (SNP) OR TOPIC: (Polymorphism) OR TOPIC: (Polymorphisms) OR TOPIC: (genetic variant) OR TOPIC: (genetic variants)	**1,617,571**
	#2	TOPIC: (NLRP1) OR TOPIC: (NALP1) OR TOPIC: (NLR Family Pyrin Domain Containing 1) OR TOPIC: (NACHT, LRR And PYD Domains-Containing Protein 1) OR TOPIC: (NACHT leucine-rich-repeat protein 1) OR TOPIC: (death effector filament-forming Ced-4-like apoptosis) OR TOPIC: (NACHT, leucine rich repeat and PYD containing 1) OR TOPIC: (NLR family, pyrin domain containing 1) OR TOPIC: (DEFCAP) OR TOPIC: (DEFCAP-S) OR TOPIC: (DEFCAP-L)	**1,118**
	#3	#1 AND #2	**274**
Embase	#1	'single nucleotide polymorphism' OR 'polymorphism, single nucleotide'	**141,613**
	#2	'nlrp1' OR 'nalp1' OR 'nlr family pyrin domain containing 1' OR 'nacht, lrr and pyd domains-containing protein 1'	**566**
	#3	#2 AND #3	**70**
CNKI	#1	NALP1; NLRP1; 基因多态性	**9**
WANFANG	#1	NALP1; NLRP1; 基因多态性	**32**
OVID	#1	('NLRP1' or 'NALP1' or 'NLR Family Pyrin Domain Containing 1' or ('NACHT, LRR and PYD Domains-Containing Protein 1')).af.	**2,027**
	#2	limit #1 to human [Limit not valid in Books@Ovid,Journals@Ovid,Your Journals@Ovid; records were retained]	**1,720**
	#3	('single nucleotide polymorphism' or 'polymorphism, single nucleotide' or 'polymorphism' or 'SNP' or 'mutation').af.	**2,653,055**
	#4	limit #3 to human [Limit not valid in Books@Ovid,Journals@Ovid,Your Journals@Ovid; records were retained]	**1,926,099**
	#5	#2 AND #4	**731**
	#6	'vitiligo'.af.	**31,569**
	#7	limit #6 to human [Limit not valid in Books@Ovid,Journals@Ovid,Your Journals@Ovid; records were retained]	**28,931**
	#8	('autoimmune diseases' or 'Disease, Autoimmune' or 'Diseases, Autoimmune' or 'Autoimmune Disease').af.	**349,179**
	#9	limit #8 to human [Limit not valid in Books@Ovid,Journals@Ovid,Your Journals@Ovid; records were retained]	**297,804**
	#10	#7 OR #9	**321,386**
	#11	#5 AND #10	**345**
Scopus	#1	(ALL ("NLRP1") OR ALL ("NALP1") OR ALL ("NLR Family Pyrin Domain Containing 1") OR ALL ("NACHT, LRR And PYD Domains-Containing Protein 1"))	**2,063**
	#2	(ALL("single nucleotide polymorphism") OR ALL ("single nucleotide polymorphism") OR ALL ("SNP") OR ALL ("mutation"))	**2,331,599**
	#3	(ALL("vitiligo") OR ALL ("autoimmune diseases") OR ALL ("Disease, Autoimmune") OR ALL ("Autoimmune Disease") OR ALL ("Diseases, Autoimmune"))	**260,096**
	#4	#1 AND #2	**1,064**
	#5	#3 AND #4	**474**
Cochrane	#1	NLRP1 or NALP1 or NLR Family Pyrin Domain Containing 1 or NACHT, LRR and PYD Domains-Containing Protein 1 (Word variations have been searched)	**7**
	#2	single nucleotide polymorphism or polymorphism or SNP or mutation	**13,706**
	#3	#1 AND #2	**5**

WOS, Web of Science; Embase, ExcerptaMedica Database; CNKI, China National Knowledge Infrastructure.

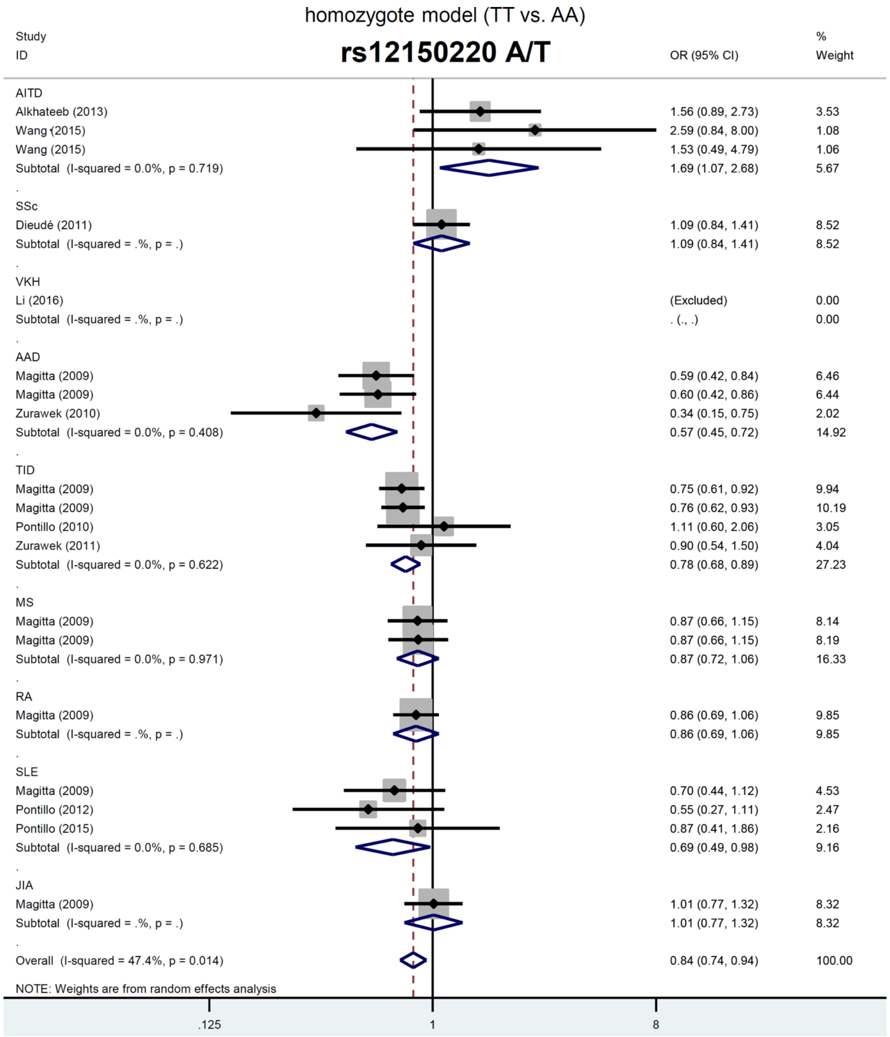

Supplementary Figure 1: Forest plots of stratified analyses by disease type for NLRP1 rs12150220, and susceptibility to vitiligo-associated autoimmune diseases under homozygote model.

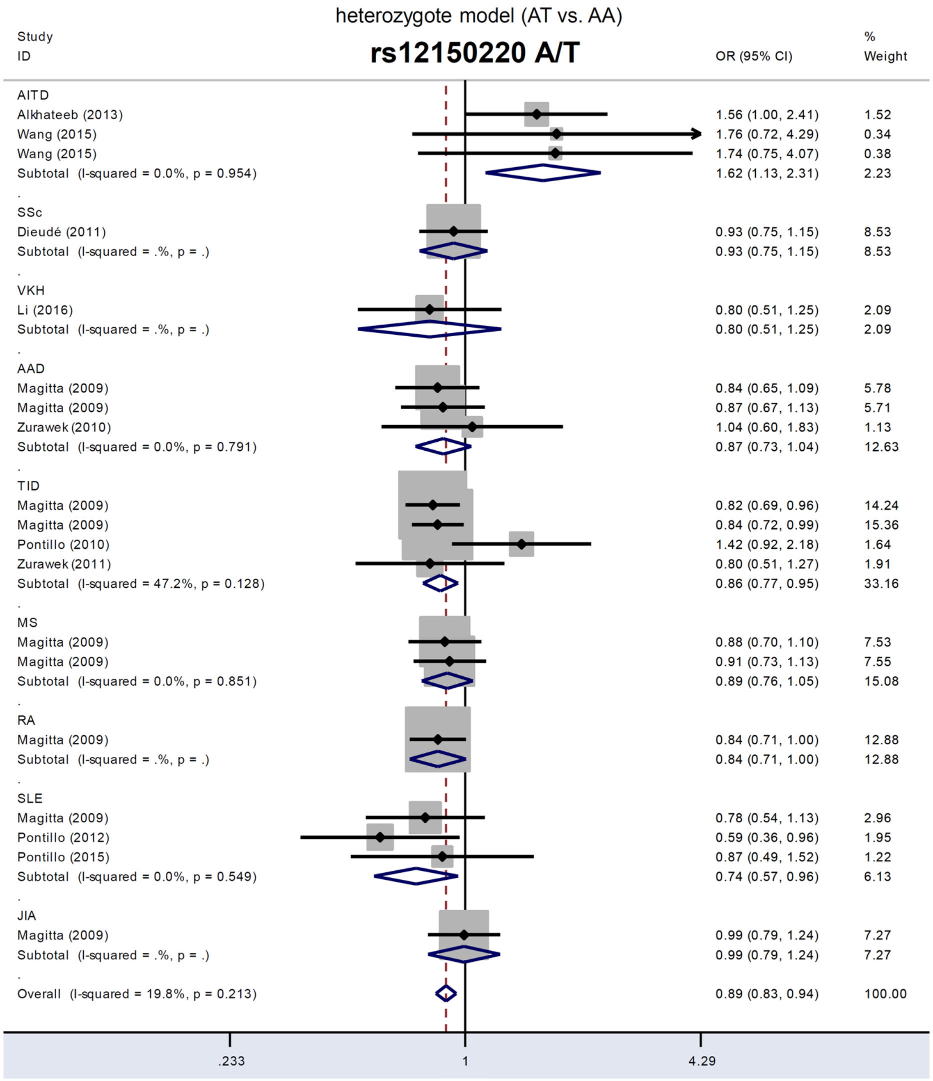

Supplementary Figure 2: Forest plots of stratified analyses by disease type for NLRP1 rs12150220, and susceptibility to vitiligo-associated autoimmune diseases under heterzygote model.

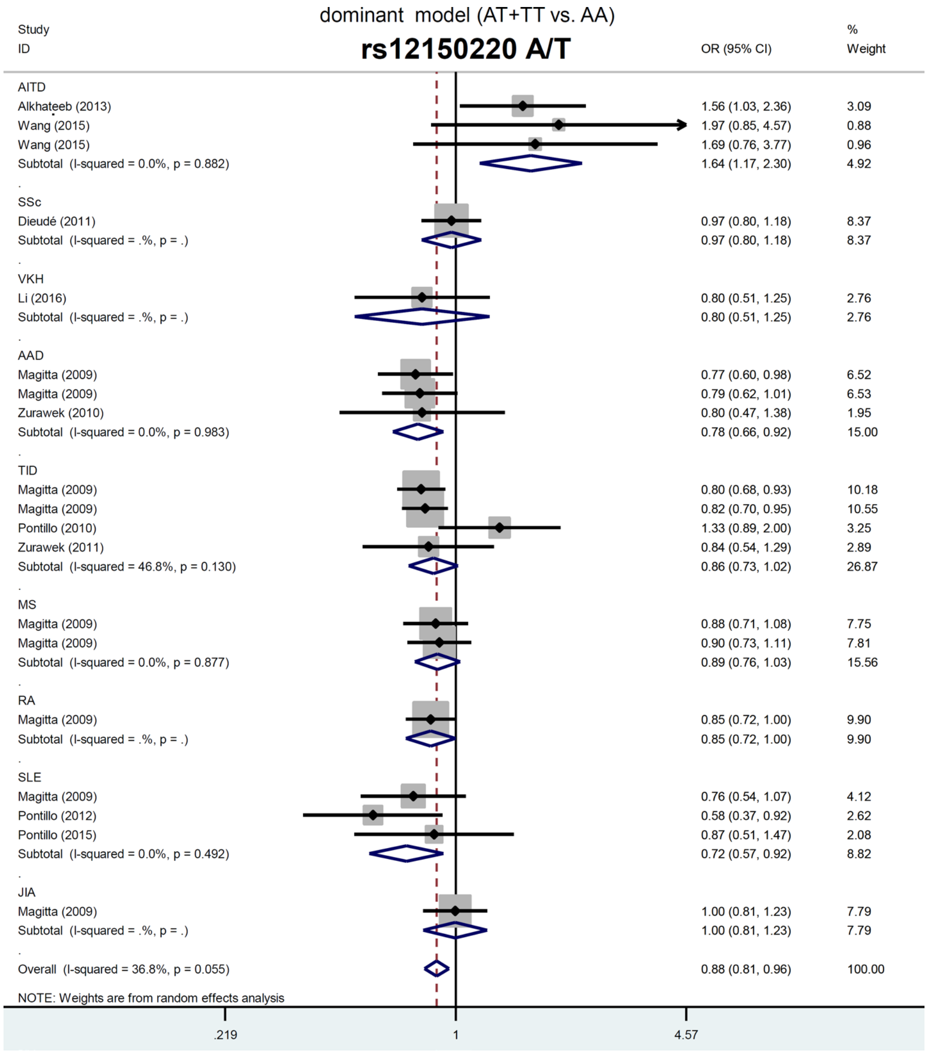

Supplementary Figure 3: Forest plots of stratified analyses by disease type for NLRP1 rs12150220, and susceptibility to vitiligo-associated autoimmune diseases under dominant model.

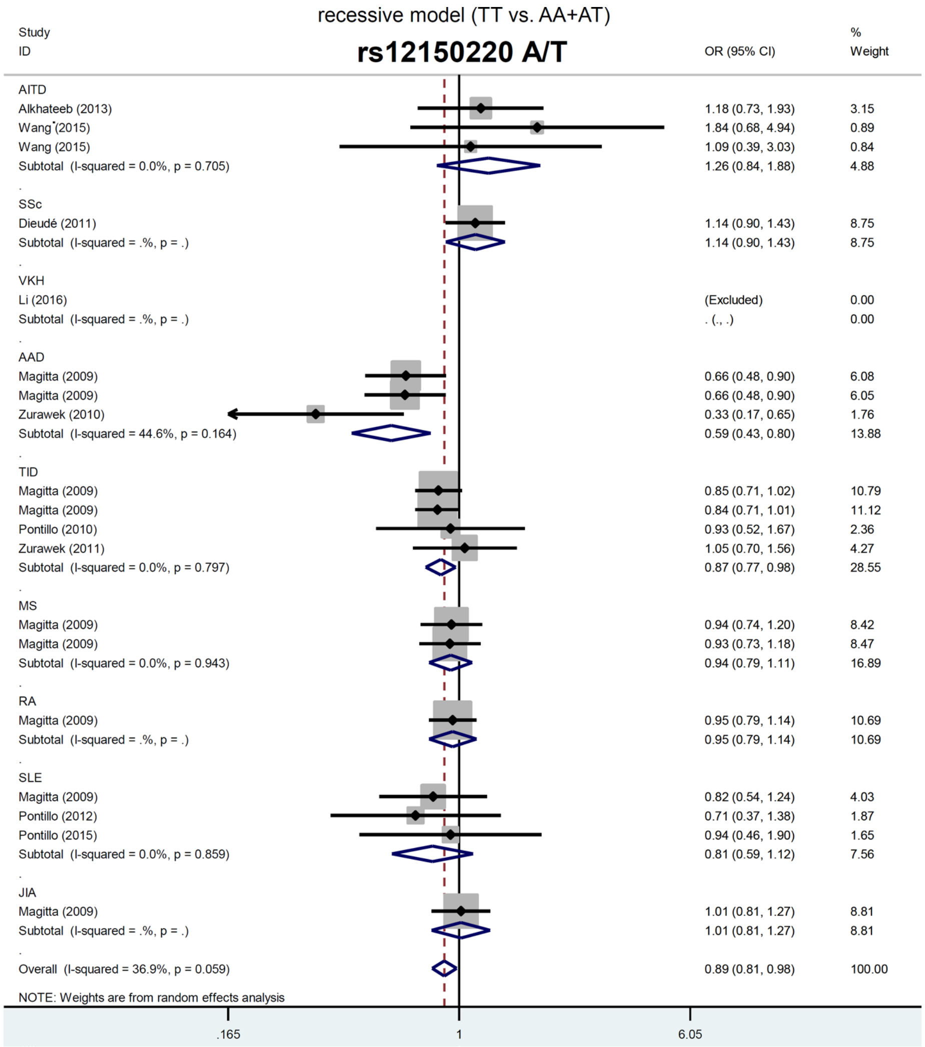

Supplementary Figure 4: Forest plots of stratified analyses by disease type for NLRP1 rs12150220 and susceptibility to vitiligo-associated autoimmune diseases under recessive model.

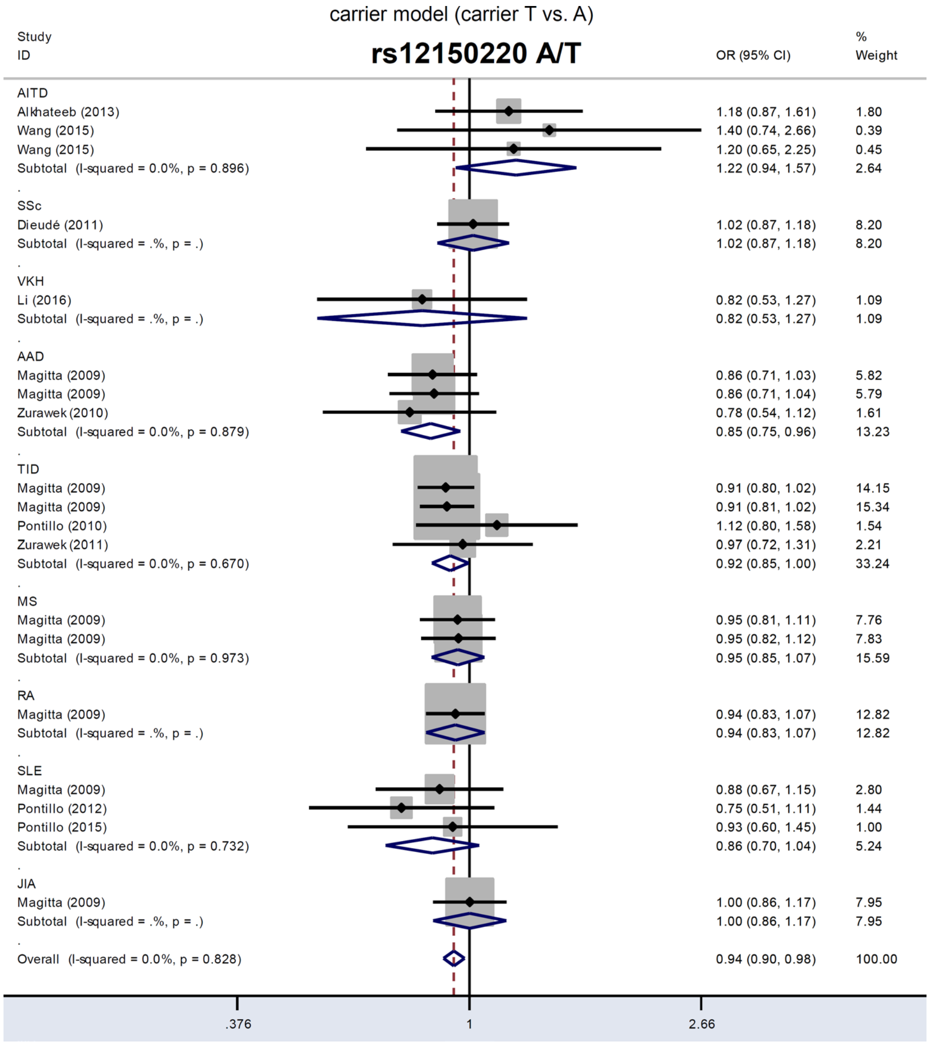

Supplementary Figure 5: Forest plots of stratified analyses by disease type for NLRP1 rs12150220, and susceptibility to vitiligo-associated autoimmune diseases under carrier model.

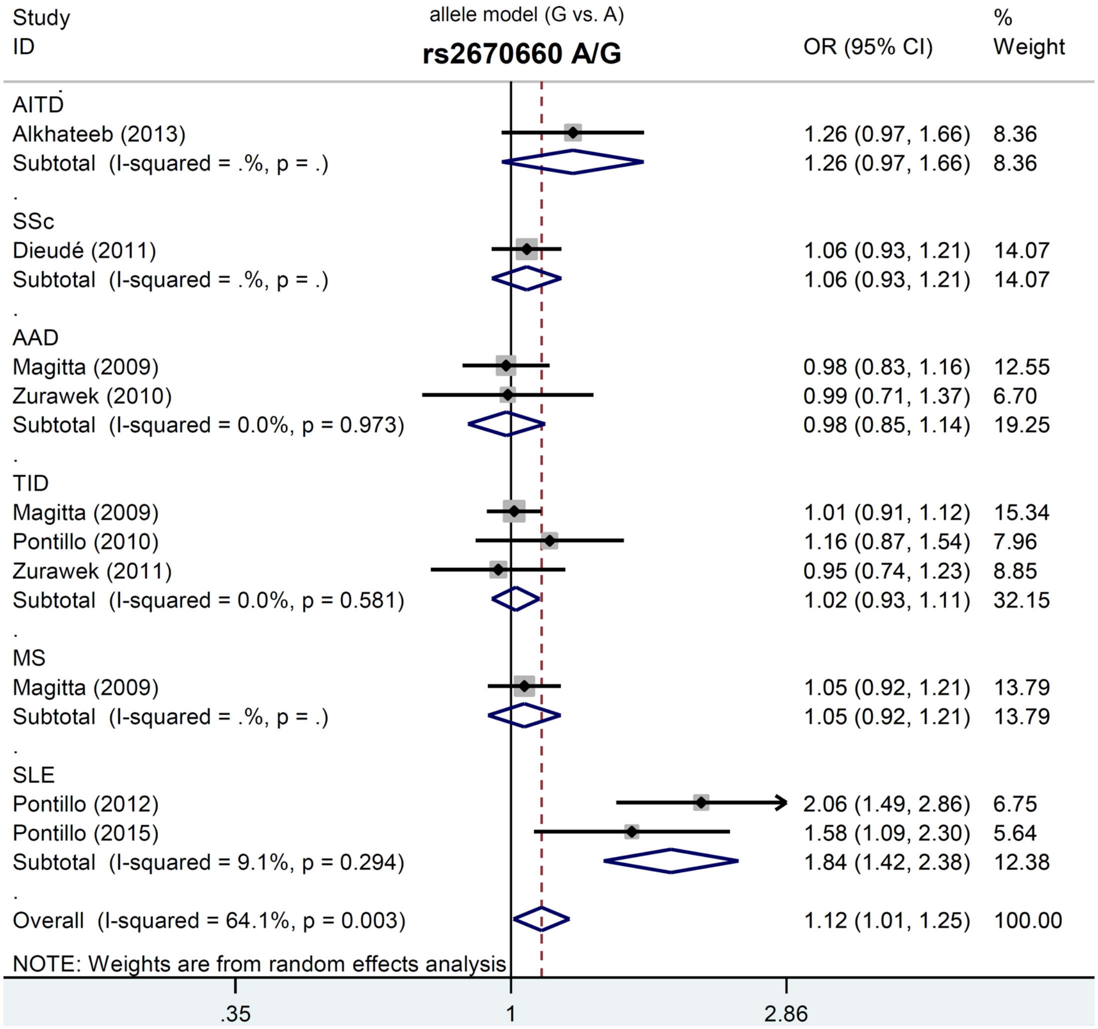

Supplementary Figure 6: Forest plots of stratified analyses by disease type for NLRP1 rs2670660, and susceptibility to vitiligo-associated autoimmune diseases under allele model.

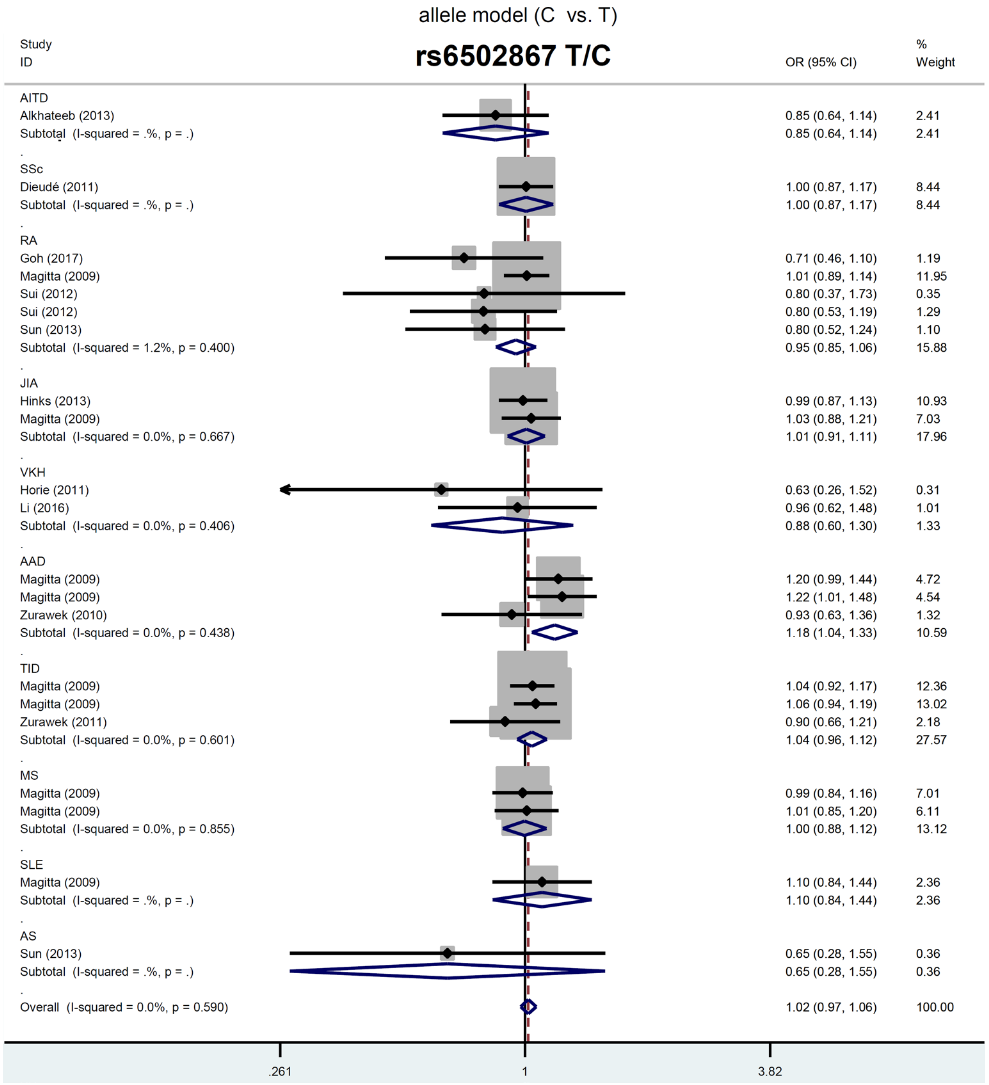

Supplementary Figure 7: Forest plots of stratified analyses by disease type for NLRP1 rs6502867, and susceptibility to vitiligo-associated autoimmune diseases under allele model.
