# Supplementary material for: Meta-analysis of the association between NLRP1 polymorphisms and the susceptibility to vitiligo and associated autoimmune diseases
Source: Oncotarget. 2017 Sep 22;8(50):88179–88. doi: 10.18632/oncotarget.21165 (PMC5675702; doi:10.18632/oncotarget.21165)
Supplement: Supplementary file 3 [file oncotarget-08-88179-s003.docx]

Supplementary Table 2: Characteristics of included case-control studies

| **First author** | **Year** | **Ethnicity** | **SNP** | **case** | | | **group** | **control** | | | **Source of control** | ***P*_HWE_** | **Genotyping method** |
| --- | --- | --- | --- | --- | --- | --- | --- | --- | --- | --- | --- | --- | --- |
|  |  |  |  | **XX** | **XY** | **YY** |  | **XX** | **XY** | **YY** |  |  |  |
| **Alkhateeb** | 2013 | Asian | rs12150220 | 54 | 112 | 41 | AITD | 78 | 104 | 38 | PB | 0.74 | PCR-RFLP |
|  |  |  | rs2670660 | 50 | 113 | 44 | AITD | 71 | 110 | 39 | PB | 0.75 |  |
|  |  |  | rs6502867 | 103 | 86 | 18 | AITD | 95 | 105 | 20 | PB | 0.23 |  |
| **Alkhateeb** | 2010 | Asian | rs12150220 | 6 | 14 | 6 | GV | 14 | 21 | 26 | PB | **0.03** | SNaPshot Multiplex Kit |
|  |  |  | rs2670660 | 5 | 13 | 8 | GV | 24 | 22 | 15 | PB | **0.04** |  |
|  |  |  | rs6502867 | 12 | 13 | 1 | GV | 33 | 23 | 5 | PB | 0.73 |  |
| **Dieudé** | 2011 | Caucasian | rs12150220 | 279 | 408 | 181 | SSc | 303 | 477 | 181 | PB | 0.78 | competitive allele specific PCR |
|  |  |  | rs2670660 | 269 | 403 | 185 | SSc | 302 | 450 | 183 | PB | 0.51 |  |
|  |  |  | rs6502867 | 486 | 315 | 69 | SSc | 511 | 379 | 56 | PB | 0.19 |  |
| **Dwivedi** | 2013 | Asian | rs12150220 | 216 | 150 | 26 | active GV | 400 | 205 | 40 | PB | 0.05 | TaqMan assay |
|  |  |  | rs12150220 | 82 | 52 | 11 | stable GV | 400 | 205 | 40 | PB | 0.05 |  |
|  |  |  | rs2670660 | 77 | 189 | 126 | active GV | 229 | 291 | 125 | PB | 0.06 | PCR-RFLP |
|  |  |  | rs2670660 | 59 | 62 | 24 | stable GV | 229 | 291 | 125 | PB | 0.06 |  |
|  |  |  | rs6502867 | 129 | 184 | 79 | active GV | 312 | 260 | 73 | PB | 0.10 |  |
|  |  |  | rs6502867 | 70 | 55 | 20 | stable GV | 312 | 260 | 73 | PB | 0.10 |  |
| **Goh** | 2017 | Asian | rs6502867 | 463 | 37 | 0 | RA | 449 | 51 | 0 | PB | 0.23 | TaqMan assay |
| **Hinks** | 2013 | Caucasian | rs6502867 | 416 | 249 | 41 | JIA | 3038 | 1863 | 299 | PB | 0.55 | Sequenom iPlex MassARRAY platform |
| **Horie** | 2011 | Asian | rs6502867 | 158 | 8 | 0 | VKH | 173 | 12 | 1 | PB | 0.14 | gene sequencing |
| **Jin** | 2007 | Caucasian | rs6502867 | 45 | 18 | 3 | GV | 46 | 39 | 8 | PB | 0.95 | SNaPshot Multiplex Kit |
|  |  |  | rs2670660 | 9 | 34 | 23 | GV | 29 | 37 | 27 | PB | 0.05 |  |
| **Li** | 2016 | Asian | rs6502867 | 343 | 35 | 0 | VKH | 515 | 55 | 0 | PB | 0.23 | PCR-RFLP+gene sequencing |
|  |  |  | rs12150220 | 336 | 33 | 0 | VKH | 506 | 62 | 0 | PB | 0.17 |  |
| **Magitta** | 2009 | Caucasian | rs12150220 | 109 | 173 | 51 | AAD | 617 | 1164 | 488 | PB | 0.16 | TaqMan allelic discrimination assay+gene sequencing |
|  |  |  | rs12150220 | 346 | 533 | 205 | TID | 617 | 1164 | 488 | PB | 0.16 |  |
|  |  |  | rs12150220 | 150 | 249 | 103 | MS | 617 | 1164 | 488 | PB | 0.16 |  |
|  |  |  | rs12150220 | 104 | 166 | 49 | AAD | 878 | 1611 | 688 | PB | 0.32 |  |
|  |  |  | rs12150220 | 340 | 525 | 202 | TID | 878 | 1611 | 688 | PB | 0.32 |  |
|  |  |  | rs12150220 | 143 | 238 | 98 | MS | 878 | 1611 | 688 | PB | 0.32 |  |
|  |  |  | rs12150220 | 270 | 418 | 181 | RA | 878 | 1611 | 688 | PB | 0.32 |  |
|  |  |  | rs12150220 | 51 | 73 | 28 | SLE | 878 | 1611 | 688 | PB | 0.32 |  |
|  |  |  | rs12150220 | 138 | 251 | 109 | JIA | 878 | 1611 | 688 | PB | 0.32 |  |
|  |  |  | rs2670660 | 102 | 184 | 47 | AAD | 733 | 1157 | 379 | PB | 0.03 | TaqMan allelic discrimination assay |
|  |  |  | rs2670660 | 354 | 539 | 191 | TID | 733 | 1157 | 379 | PB | 0.03 |  |
|  |  |  | rs2670660 | 158 | 252 | 92 | MS | 733 | 1157 | 379 | PB | 0.03 |  |
|  |  |  | rs6502867 | 180 | 133 | 20 | AAD | 1359 | 792 | 118 | PB | 0.85 |  |
|  |  |  | rs6502868 | 638 | 386 | 60 | TID | 1359 | 792 | 118 | PB | 0.85 |  |
|  |  |  | rs6502869 | 313 | 153 | 36 | MS | 1359 | 792 | 118 | PB | 0.85 |  |
|  |  |  | rs6502867 | 168 | 124 | 19 | AAD | 1888 | 1075 | 161 | PB | 0.62 |  |
|  |  |  | rs6502867 | 615 | 372 | 58 | TID | 1888 | 1075 | 161 | PB | 0.62 |  |
|  |  |  | rs6502867 | 256 | 125 | 30 | MS | 1888 | 1075 | 161 | PB | 0.62 |  |
|  |  |  | rs6502867 | 549 | 328 | 43 | RA | 1888 | 1075 | 161 | PB | 0.62 |  |
|  |  |  | rs6502867 | 88 | 52 | 10 | SLE | 1888 | 1075 | 161 | PB | 0.62 |  |
|  |  |  | rs6502867 | 295 | 159 | 32 | JIA | 1888 | 1075 | 161 | PB | 0.62 |  |
| **Pontillo** | 2010 | Mixed | rs12150220 | 78 | 92 | 26 | TID | 90 | 75 | 27 | PB | 0.08 | TaqMan allelic discrimination assay+gene sequencing |
|  |  |  | rs2670660 | 55 | 90 | 51 | TID | 60 | 90 | 42 | PB | 0.45 |  |
| **Pontillo** | 2012 | Mixed | rs12150220 | 73 | 54 | 17 | SLE | 59 | 74 | 25 | PB | 0.82 | TaqMan allelic discrimination assay+gene sequencing |
|  |  |  | rs2670660 | 21 | 75 | 48 | SLE | 49 | 87 | 22 | PB | 0.09 |  |
| **Pontillo** | 2015 | Mixed | rs12150220 | 39 | 37 | 14 | SLE | 63 | 69 | 26 | PB | 0.34 | TaqMan allelic discrimination assay+gene sequencing |
|  |  |  | rs2670660 | 17 | 48 | 22 | SLE | 49 | 87 | 22 | PB | 0.09 |  |
| **Sui** | 2012 | Asian | rs6502867 | 176 | 12 | 0 | RA (Chengdu) | 174 | 15 | 0 | PB | 0.57 | TaqMan allele discrimination assay |
|  |  |  | rs6502867 | 712 | 44 | 0 | RA (Chongqing) | 702 | 55 | 0 | PB | 0.30 |  |
| **Sun** | 2013 | Asian | rs6502867 | 481 | 38 | 0 | RA | 474 | 45 | 1 | HB | 0.95 | MALDI-TOF MS |
|  |  |  | rs6502867 | 94 | 6 | 0 | AS | 474 | 45 | 1 | HB | 0.95 |  |
| **Wang** | 2015 | Asian | rs12150220 | 15 | 31 | 16 | AITD (GD) | 17 | 20 | 7 | PB | 0.78 | PCR-RFLP |
|  |  |  | rs12150220 | 19 | 39 | 12 | AITD (HT) | 17 | 20 | 7 | PB | 0.78 |  |
| **Xie** | 2008 | Asian | rs6502867 | 81 | 8 | 1 | vitiligo | 81 | 9 | 0 | PB | 0.62 | PCR-RFLP |
|  |  |  | rs2670660 | 66 | 22 | 2 | vitiligo | 62 | 24 | 4 | PB | 0.40 |  |
| **Zurawek** | 2011 | Caucasian | rs6502867 | 133 | 76 | 12 | TID | 148 | 87 | 19 | PB | 0.22 | PCR-RFLP |
|  |  |  | rs12150220 | 53 | 106 | 62 | TID | 53 | 132 | 69 | PB | 0.49 |  |
|  |  |  | rs2670660 | 69 | 98 | 54 | TID | 70 | 125 | 59 | PB | 0.82 |  |
| **Zurawek** | 2010 | Caucasian | rs6502867 | 58 | 39 | 4 | AAD | 148 | 87 | 19 | PB | 0.22 | PCR-RFLP |
|  |  |  | rs12150220 | 25 | 65 | 11 | AAD | 53 | 132 | 69 | PB | 0.49 |  |
|  |  |  | rs2670660 | 25 | 56 | 20 | AAD | 70 | 125 | 59 | PB | 0.82 |  |

SNP: Single Nucleotide Polymorphism; X, major allele; Y, minor allele; AITD, autoimmune thyroid disease; SSc, systemic sclerosis; GV, generalized vitiligo; JIA, juvenile idiopathic arthritis; VKH, Vogt-Koyanagi-Harada disease; AAD, autoimmune Addison’s disease; TID, type 1 diabetes; RA, rheumatoid arthritis; MS, multiple sclerosis; SLE, systemic lupus erythematosus; AS, ankylosing spondylitis; GD, Graves'disease; HT, Hashimoto' thyroiditis; PB, population-based; HB, hospital-based; PCR-RFLP: Polymerase chain reaction-Restriction fragment length polymorphism; MALDI-TOF MS: matrix-assisted laser desorption/ionization time-of-flight mass spectrometry; *P*_HWE_: *P* value of Hardy-Weinberg equilibrium.
