# Supplementary material for: Meta-analysis of the association between NLRP1 polymorphisms and the susceptibility to vitiligo and associated autoimmune diseases
Source: Oncotarget. 2017 Sep 22;8(50):88179–88. doi: 10.18632/oncotarget.21165 (PMC5675702; doi:10.18632/oncotarget.21165)
Supplement: Supplementary file 4 [file oncotarget-08-88179-s004.docx]

Supplementary Table 7: Pooled analyses of the association between *NLRP1* rs6502867 polymorphism and susceptibility to vitiligo-associated autoimmune diseases.

| **Genetic models** | **Stratification** | **case/control (N)** | **OR [95 % CI]** | ***P_association_*** |
| --- | --- | --- | --- | --- |
| **C vs. T** | overall | 9,954/35,667 (21) | 1.02[0.97~1.06] | 0.427 |
|  | Asian | 2,814/3,462 (8) | 0.81[0.69~0.95] | **0.010** |
|  | Caucasian | 7,140/32,205 (13) | 1.04[0.99~1.08] | 0.122 |
|  | PB | 9,335/34,627 (19) | 1.02[0.98~1.07] | 0.340 |
|  | AAD | 745/5,647 (3) | 1.18[1.04~1.33] | **0.012** |
|  | TID | 2,350/5,647 (3) | 1.04[0.96~1.12] | 0.384 |
|  | RA | 2,883/5,090 (5) | 0.95[0.85~1.06] | 0.360 |
| **CC vs. TT** | overall | 8,132/33,651 (17) | 1.12[1.00~1.25] | 0.053 |
|  | Asian | 992/1,446 (4) | 0.78[0.41~1.49] | 0.457 |
|  | Caucasian | 7,140/32,205 (13) | 1.13[1.01~1.27] | **0.035** |
|  | PB | 8,835/34,127 (15) | 1.12[1.00~1.26] | 0.050 |
|  | AAD | 745/5,647 (3) | 1.18[0.84~1.65] | 0.342 |
|  | TID | 2,350/5,647 (3) | 1.05[0.85~1.31] | 0.630 |
|  | RA | 2,383/4,590 (2) | 0.91[0.64~1.28] | 0.576 |
| **TC vs. TT** | overall | 9,954/35,667 (21) | 0.99[0.93~1.04] | 0.596 |
|  | Asian | 2,814/3,462 (8) | 0.79[0.66~0.94] | **0.010** |
|  | Caucasian | 7,140/32,205 (13) | 1.01[0.95~1.07] | 0.780 |
|  | PB | 9,335/34,627 (19) | 0.99[0.94~1.05] | 0.707 |
|  | AAD | 745/5,647 (3) | 1.27[1.08~1.49] | **0.004** |
|  | TID | 2,350/5,647 (3) | 1.04[0.94~1.16] | 0.405 |
|  | RA | 2,883/5,090 (5) | 0.96[0.84~1.09] | 0.513 |
| **TC+CC vs. TT** | overall | 9,954/35,667 (21) | 1.00[0.95~1.05] | 0.959 |
|  | Asian | 2,814/3,462 (8) | 0.79[0.66~0.94] | **0.008** |
|  | Caucasian | 7,140/32,205 (13) | 1.02[0.97~1.08] | 0.375 |
|  | PB | 9,335/34,627 (19) | 1.01[0.95~1.06] | 0.825 |
|  | AAD | 745/5,647 (3) | 1.25[1.07~1.46] | **0.004** |
|  | TID | 2,350/5,647 (3) | 1.05[0.95~1.15] | 0.371 |
|  | RA | 2,883/5,090 (5) | 0.95[0.84~1.08] | 0.420 |
| **CC vs. TT+TC** | overall | 8,132/33,651 (17) | 1.12[1.00~1.25] | **0.043** |
|  | Asian | 992/1,446 (4) | 0.89[0.48~1.65] | 0.707 |
|  | Caucasian | 7,140/32,205 (13) | 1.13[1.01~1.27] | **0.033** |
|  | PB | 8,835/34,127 (15) | 1.12[1.00~1.26] | **0.041** |
|  | AAD | 745/5,647 (3) | 1.08[0.77~1.50] | 0.666 |
|  | TID | 2,350/5,647 (3) | 1.04[0.84~1.28] | 0.731 |
|  | RA | 2,383/4,590 (2) | 0.89[0.63~1.25] | 0.507 |
| **carrier C vs. T** | overall | 9,954/35,667 (21) | 1.00[0.96~1.05] | 0.856 |
|  | Asian | 2,814/3,462 (8) | 0.82[0.70~0.98] | 0.026 |
|  | Caucasian | 7,140/32,205 (13) | 1.02[0.97~1.07] | **0.033** |
|  | PB | 9,335/34,627 (19) | 1.01[0.96~1.06] | 0.743 |
|  | AAD | 745/5,647 (3) | 1.14[0.99~1.31] | 0.666 |
|  | TID | 2,350/5,647 (3) | 1.03[0.94~1.13] | 0.731 |
|  | RA | 2,883/5,090 (5) | 0.95[0.84~1.07] | 0.401 |

PB, population-based control; AAD, Autoimmune Addison’s disease; TID, Type 1 diabetes; RA, Rheumatoid arthritis;

OR, odds ratio; CI, confidence interval; *P_association_*, *P* value of Association Test; N, Number of included case-control studies.
